# Job vacancy at the European Centre for Disease Prevention and Control

**DOI:** 10.2807/1560-7917.ES.2018.23.32.1808091

**Published:** 2018-08-09

**Authors:** Eurosurveillance editorial team

**Affiliations:** 1 European Centre for Disease Prevention and Control (ECDC), Stockholm, Sweden

The European Centre for Disease Prevention and Control (ECDC) invites applications for the following positions:

**Disease Programme Coordination Manager**

The application deadline expires on 03 September 2018. 

**Financial assistant**

The application deadline expires on 03 September 2018.

For more information on either position, please visit the ECDC website: https://ecdc.europa.eu/en/work-us/vacancies?f%5B0%5D=deadline_date%3A1&f%5B1%5D=vacancy_type%3A1700

